# Role of Human Leukocyte Antigen Allele Sharing in Human Papillomavirus Infection Transmission Among Heterosexual Couples: Findings From the HITCH Cohort Study^[Author-notes jiac115-FM1]^

**DOI:** 10.1093/infdis/jiac115

**Published:** 2022-04-01

**Authors:** Karolina Louvanto, Prativa Baral, Ann Burchell, Agnihotram Ramanakumar, Mariam El-Zein, Pierre Paul Tellier, Francois Coutlée, Michel Roger, Eduardo L Franco

**Affiliations:** Division of Cancer Epidemiology, McGill University, Montreal, Quebec, Canada; Department of Obstetrics and Gynaecology, Faculty of Medicine and Health Technology, Tampere University, Tampere, Finland; Department of Obstetrics and Gynaecology, Tampere University Hospital, Tampere, Finland; Division of Cancer Epidemiology, McGill University, Montreal, Quebec, Canada; Ontario HIV Treatment Network, Toronto, Ontario, Canada; Division of Cancer Epidemiology, McGill University, Montreal, Quebec, Canada; Division of Cancer Epidemiology, McGill University, Montreal, Quebec, Canada; Department of Family Medicine, McGill University, Montreal, Quebec, Canada; Division of Cancer Epidemiology, McGill University, Montreal, Quebec, Canada; Centre de recherche du Centre hospitalier de l'Université de Montréal et Département de microbiologie, infectiologie et immunologie, Université de Montréal, Montreal, Quebec, Canada; Centre de recherche du Centre hospitalier de l'Université de Montréal et Département de microbiologie, infectiologie et immunologie, Université de Montréal, Montreal, Quebec, Canada; Division of Cancer Epidemiology, McGill University, Montreal, Quebec, Canada

**Keywords:** HPV, HLA, transmission, heterosexual, cervical, couple

## Abstract

**Background:**

Human leukocyte antigen (HLA) polymorphism influences innate and adaptive immune responses. Among heterosexual couples in the HPV Infection and Transmission Among Couples Through Heterosexual Activity (HITCH) cohort study, we examined whether allele sharing in a couple predicted the partners’ infections with the same human papillomavirus (HPV) type.

**Methods:**

We tested genital samples from 271 couples for 36 HPV genotypes by polymerase chain reaction. We used direct DNA sequencing to type HLA-B07, -DRB1, -DQB1 and -G. Generalized estimating equations were used to examine the associations between the extent of allele sharing and HPV type concordance in which at least 1 of the partners was HPV positive.

**Results:**

We identified 106 different HLA alleles. The most common HLA alleles among couples were G*01:01:01 (95.6%), G*01:01:02 (60.1%), DQB1*03:01 (57.2%), and DRB1*07:01 (46.9%). Allele sharing was as follows: 19.6% shared none, 43.2% shared 1 only, 25.1% shared 2, and 12.5% shared 3–5. Irrespective of HLA class, grouped or in combination, the extent of allele sharing was not a significant predictor of type-specific HPV concordance in a couple (odds ratio, 1.1 [95% confidence interval, .5–2.1], for 3–5 vs none).

**Conclusions:**

We found no evidence that the extent of HLA allele concordance influences the likelihood of HPV transmission in newly formed heterosexual couples.

## Teaser

Among heterosexual couples, we examined whether human leukocyte antigen (HLA) allele sharing in a couple predicted the partners’ infections with the same human papillomavirus (HPV) type. Our evaluations, however, could not provide evidence that the extent of HLA allele concordance influences the likelihood of HPV transmission.

Persistent human papillomavirus (HPV) infection causes 5% of all cancers worldwide. Nearly 10% of all female cancers, particularly cervical cancer, are caused by HPV [1, 2]. HPV is a common sexually transmitted infection in young women and men. Genital HPV infections are mostly transient and clear spontaneously in 12–24 months after acquisition [3, 4]. Characteristics that affect transmission of HPV infection in heterosexual couples are mostly behavioral, hormonal, or virus related [5]. Host genetic variation, particularly human leukocyte antigen (HLA) polymorphism, is an important driver in HPV-associated cervical carcinogenesis [6]. However, little is known about the role of HLA polymorphism in the transmission of HPV within sexually active couples.

HLA genes influence innate and adaptive immune responses. The different HLA genes are classified into class I (HLA-A, -B, and -C) and class II (HLA-DRB and -DQB) alleles and the class Ib (HLA-G) alleles [7]. These different HLA alleles cluster in various genes that mediate antigen presentation and cell-mediated immune response by facilitating the recognition and clearance of virus-infected cells [8]. Sharing of HLA-B alleles seems to facilitate transmission of human immunodeficiency virus type 1 (HIV-1) between serologically discordant heterosexual partners [9]. Likewise, risk of vertical transmission of HIV-1 is increased with HLA allele concordance between mother and child [10]. On the other hand, concordance of HLA-G alleles between mother and infant did not seem to affect vertical transmission of HPV infection between the mother and her neonate [11].

Among heterosexual couples in the HPV Infection and Transmission Among Couples Through Heterosexual Activity (HITCH) cohort study, we examined whether HLA-B07, -DRB1, -DQB1 and -G allele sharing in a couple predicted the partners’ infections with the same HPV type. Our hypothesis was that HLA allele concordance would facilitate transmission of HPV infection by lowering the likelihood of HLA-mediated rejection of exfoliated HPV-harboring cells exchanged during sex.

## MATERIALS AND METHODS

The HITCH study is a longitudinal cohort investigation conducted at McGill University from May 2005 to January 2011 that enrolled young female university and junior college students (aged 18–24 years) and their male partners (at least 18 years old) whose sexual activity was initiated within the previous 6 months prior to enrollment. Study procedures have been described previously [12, 13]. In brief, participants completed computerized self-administered questionnaires and provided biologic samples for HPV assessment. Participants were asked to abstain from oral, vaginal, and anal sex for 24 hours prior to clinic visits. Women self-collected vaginal specimens using a Dacron swab and a clinic nurse collected penile and scrotal samples at each clinic visit for the men. The Linear Array HPV genotyping assay (Roche Molecular Systems) was used to detect 36 mucosal HPV genotypes (6, 11, 16, 18, 26, 31, 33, 34, 35, 39, 40, 42, 44, 45, 51, 52, 53, 54, 56, 58, 59, 61, 62, 66, 67, 68, 69, 70, 71, 72, 73, 81, 82, 83, 84, and 89). β-globin DNA was coamplified to assess DNA integrity. The ethical review committees of McGill University, Concordia University, and Université de Montréal approved the study. All participants provided written informed consent.

### HLA Typing

Purified DNA from enrollment genital specimens collected at enrollment was used for HLA typing. HLA class I (B*07) was typed using polymerase chain reaction with sequence-specific primers as described previously [14]. HLA-DQB1 and -DRB1 alleles were determined by sequence-based typing with the Allele SEQR DRB1 and Allele SEQR DQB1 assays (Abbott Molecular Diagnostics), respectively. HLA-G alleles were determined through direct DNA sequencing of the nucleotide regions encompassing the HLA-G exons 2–4 (1718 bp) as described previously [15]. The HLA-G 3ʹ untranslated region (UTR) genetic variants including the 14 bp deletion/insertion polymorphism was determined by DNA sequencing according to the protocol [16].

### Statistical Analysis

Stata 12.0 software (StataCorp, College Station, Texas) was used for all statistical analyses. Genital HPV types, belonging to the genus *Alphapapillomavirus*, were categorized into 3 subgenera based on their phylogenetic relatedness, as follows: subgenus 1: HPV types that cause genital warts or asymptomatic infections (6, 11, 40, 42, 44, and 54); subgenus 2: HPV types that have possible, probable, or proven carcinogenic effects (16, 18, 26, 31, 33, 34, 35, 39, 45, 51, 52, 53, 56, 58, 59, 66, 67, 68, 69, 70, 73, and 82); and subgenus 3: HPV types that cause mostly commensal vaginal infections (61, 62, 71, 72, 81, 83, 84, and 89) [17]. We combined the results for HPV type-specific positivity at enrollment and 4-month follow-up visits to derive a period prevalence estimate of HPV type-specific infections in females and males. Type-specific HPV concordance among partners in each couple (dyad) was restricted to couples positive for that type. For the sake of statistical precision, we only considered HLA alleles that were present in at least 5% of female or male participants. We analyzed type-specific HPV concordance overall and grouped by HPV phylogenetic groups among couples according to the extent of allele sharing by HLA class and for all HLA polymorphisms combined. We used unconditional logistic regression for HLA level sharing and generalized estimation equations with logistic link for grouped HPV types.

## RESULTS

We identified a total of 106 different HLA alleles among the evaluable 271 female and male participants. Table 1 shows the alleles that were prevalent in at least 5% of the participants. Since 3 possibilities exist for each individual—that is, allele is absent, heterozygous (or heteroallelic), or homozygous (homoallelic)—there are 3 × 3 = 9 possible combinations of allele sharing per couple. The most common HLA alleles among couples were G*01:01:01 (95.6%), G*01:01:02 (60.1%), DQB1*03:01 (57.2%), and DRB1*07:01 (46.9%), followed by other alleles that were present between 0% (not shown in table) and 37.6% among the couples. The HLA-G*14bp deletion occurred in 86.3% of the couples and the different HLA-G 3ʹUTR single-nucleotide polymorphisms (SNPs) ranged between 8.5% and 94.8%.

**Table 1.** Prevalence of Different Human Leukocyte Antigen (HLA) Alleles^a^ and the HLA-G 3ʹ Untranslated Region Single-Nucleotide Polymorphisms Among 271 Heterosexual Couples in the Human Papillomavirus Infection and Transmission Among Couples Through Heterosexual Activity (HITCH) Cohort Study

	HLA Genotype Concordance Among Couples (Frequencies Female/Male)									
HLA	Ab/Ab	Ab/Het	Ab/Hom	Het/Ab	Het/Het	Het/Hom	Hom/Ab	Hom/ Het	Hom/Hom	Total
B*07	169	44	0	43	14	0	1	0	0	271
DRB1*01:01	222	20	1	26	2	0	0	0	0	271
DRB1*03:01	180	33	2	43	10	0	3	0	0	271
DRB1*04:01	216	30	0	21	3	0	1	0	0	271
DRB1*04:04	237	18	0	15	0	0	1	0	0	271
DRB1*07:01	144	63	3	43	16	1	1	0	0	271
DRB1*11:01	208	27	4	27	3	0	2	0	0	271
DRB1*11:04	224	20	2	21	3	0	1	0	0	271
DRB1*15:01	177	41	2	37	11	0	3	0	0	271
DRB1*16:01	243	13	1	11	1	1	0	0	1	271
DQB1*02:01	181	37	2	40	8	0	3	0	0	271
DQB1*02:02	174	50	4	32	9	1	1	0	0	271
DQB1*03:01	116	56	11	54	17	6	8	2	1	271
DQB1*03:02	175	34	5	44	7	1	5	0	0	271
DQB1*03:03	233	18	0	17	2	0	0	1	0	271
DQB1*04:02	242	9	0	16	2	0	2	0	0	271
DQB1*05:01	179	39	6	41	4	0	2	0	0	271
DQB1*05:02	237	16	1	13	1	1	1	0	1	271
DQB1*05:03	247	9	0	11	4	0	0	0	0	271
DQB1*06:02	174	36	3	40	14	0	3	1	0	271
DQB1*06:03	223	13	1	23	9	0	2	0	0	271
DQB1*06:04	235	17	0	17	2	0	0	0	0	271
G*01:01:01	12	43	16	49	69	24	15	29	14	271
G*01:01:02	108	48	8	55	29	4	12	7	0	271
G*01:01:03	218	26	0	23	3	0	1	0	0	271
G*01:03	219	27	0	25	0	0	0	0	0	271
G*01:04:01	179	42	3	33	10	0	2	2	0	271
G*01:06	213	32	1	20	5	0	0	0	0	271
G*14bp	37	40	21	41	58	16	20	23	10	266
+3001 C/T	248	4	0	9	5	0	0	0	0	266
+3003 T/C	155	30	12	43	9	2	9	3	3	266
+3010 G/C	130	5	52	7	1	5	52	4	11	267
+3027 C/A	234	17	0	12	3	0	1	0	0	267
+3035 C/T	207	32	0	23	4	0	1	0	0	267
+3142 C/G	55	22	27	25	41	31	27	28	11	267
+3187 G/A	14	14	26	15	25	47	29	38	59	267
+3196 C/G	129	32	8	34	25	9	20	8	2	267

Abbreviations: Ab, absent; Het, heterozygous; HLA, human leukocyte antigen; Hom, homozygous.

a Only alleles with >5% prevalence among female or male participants are included.

The number of couples by shared HLA alleles is shown in Table 2. To be sharing an HLA allele, the dyads both had to have at least 1 allele of a specific HLA type. The HLA alleles were grouped into different groups based on inclusiveness of the HLA classification. First, we considered sharing only for classical class I and II HLA loci (HLA-B*07, -DRB1, and -DQB1). A second grouping included sharing of nonclassical class I HLA-G loci. A third grouping considered the extent of allele sharing without distinction of HLA class. We further considered sharing of HLA-G alleles combined with the 3ʹUTR variants by creating 2 different sets as follows. The first set included the 3ʹUTR 14 bp +3142 C/G and +3187 G/A together, based on knowledge of alleles that are in linkage disequilibrium. The second HLA-G group had all the HLA-G alleles and all HLA-G 3ʹUTR SNPs. The range of shared alleles varied between 0 and 6. Allele sharing among couples with all HLAs was as follows: 19.6% shared no alleles, 43.2% shared 1 only, 25.1% shared 2, and 12.5% shared between 3 and 5.

**Table 2.** Number of Couples With Shared Human Leukocyte Antigen (HLA) Alleles or Shared HLA-G 3ʹ Untranslated Region Single-Nucleotide Polymorphisms in the Human Papillomavirus Infection and Transmission Among Couples Through Heterosexual Activity (HITCH) Cohort Study

	No. of Couples Sharing Alleles According to Inclusiveness of HLA Classification			No. of Couples Sharing HLA-G 3ʹUTR SNPs	
No. of Shared Alleles	HLA-B*07, -DRB1, -DQB1 Loci	HLA-G Loci	All HLA Loci	HLA-G: 14 bp, +3142 C/G, +3187 G/A	HLA-G: 14 bp and All 8 Different SNPs
0	160	92	53	66	65
1	64	161	117	85	77
2	42	18	67	58	46
3	5	…	28	62	33
4	…	…	5	…	38
5	…	…	1	…	11
6	…	…	…	…	1

Abbreviations: HLA, human leukocyte antigen; SNP, single-nucleotide polymorphism; UTR, untranslated region.

The type- and subgenus-specific HPV infection prevalence of the dyads is shown in Table 3. HPV-16 had the highest prevalence (22.88%), followed by HPV-84 (20.30%). Among types that were prevalent in at least 5% of the dyads, concordance varied from 26.32% for HPV-40 to 71.43% for HPV-82. Subgenus 3 had the highest concordance among partners (56.08% [95% confidence interval, 46.78%–65.39%]).

**Table 3.** Type- and Group-Specific Human Papillomavirus (HPV) Prevalence and Couple Concordance Among the 271 Couples of the HPV Infection and Transmission Among Couples Through Heterosexual Activity (HITCH) Cohort Study

Subgenus and HPV Type	F^–^/M^–^	F^–^/M^+^	F^+^/M^–^	F^+^/M^+^	Total	Prevalence in Dyads	Prevalence, %	Concordance Within Positive Dyads, % (95% CI)
Subgenus 1								
6	246	9	6	10	271	25	9.23	40.00 (23.40–59.26)
11	268	1	0	2	271	3	1.11	66.67 (20.77–93.85)
40	252	11	3	5	271	19	7.01	26.32 (11.81–48.79)
42	230	13	8	20	271	41	15.13	48.78 (34.25–63.52)
44	258	5	4	4	271	13	4.80	30.77 (12.68–57.63)
52	246	2	9	14	271	25	9.23	56.00 (37.07–73.33)
Subtotal	1500	41	30	55	1626	126	7.75	43.65 (31.63–55.57)
Subgenus 2								
16	209	15	11	36	271	62	22.88	58.06 (45.67–69.52)
18	257	6	4	4	271	14	5.17	28.57 (11.72–54.65)
26	271	0	0	0	271	0	0.00	…
31	251	4	8	8	271	20	7.38	40.00 (21.88–61.34)
33	262	4	3	2	271	9	3.32	22.22 (6.32–54.74)
34	269	2	0	0	271	2	0.74	0.00 (.00–65.76)
35	267	1	1	2	271	4	1.48	50.00 (15.00–85.00)
39	240	12	5	14	271	31	11.44	45.16 (29.16–62.23)
45	267	1	3	0	271	4	1.48	0.00 (.00–48.99)
51	229	12	6	24	271	42	15.50	57.14 (42.21–70.88)
53	234	13	8	16	271	37	13.65	43.24 (28.67–59.09)
54	248	7	3	13	271	23	8.49	56.52 (36.81–74.37)
56	246	11	7	7	271	25	9.23	28.00 (14.28–47.58)
58	245	8	5	13	271	26	9.59	50.00 (32.06–67.94)
59	248	6	5	12	271	23	8.49	52.17 (32.96–70.76)
66	226	20	8	17	271	45	16.61	37.78 (25.11–52.37)
67	243	9	10	9	271	28	10.33	32.14 (17.93–50.66)
68	260	1	4	6	271	11	4.06	54.55 (28.01–78.73)
69	271	0	0	0	271	0	0.00	…
70	269	0	0	2	271	2	0.74	100.00 (34.24–100.00)
73	249	7	6	9	271	22	8.12	40.91 (23.26–61.27)
Subtotal	5261	139	97	194	5691	430	7.56	45.12 (38.73–51.50)
Subgenus 3								
61	250	10	4	7	271	21	7.75	33.33 (17.19–54.63)
62	238	3	10	20	271	33	12.18	60.61 (43.68–75.32)
71	271	0	0	0	271	0	0.00	…
72	268	2	0	1	271	3	1.11	33.33 (6.15–79.23)
81	263	1	0	7	271	8	2.95	87.50 (52.91–97.76)
82	257	4	0	10	271	14	5.17	71.43 (45.35–88.28)
83	266	1	1	3	271	5	1.85	60.00 (23.07–88.24)
84	216	19	8	28	271	55	20.30	50.91 (38.08–63.62)
89	221	10	10	30	271	50	18.45	60.00 (46.18–72.39)
Subtotal	2250	50	33	106	2439	189	7.75	56.08 (46.78–65.39)
Any HPV	9011	230	160	355	9756	745	7.64	47.65 (43.07–52.24)

Data are presented as No. unless otherwise indicated.

Abbreviations: –, negative; +, positive; CI, confidence interval; F, female; HPV, human papillomavirus; M, male.

Under the assumption that HPV type and subgenus concordance implies transmission episodes within couples, we examined the effect of allele sharing on concordance among the 271 couples that were HPV positive. Allele prevalence could be complete (ie, both partners harboring the allele or partial) with only 1 member of the couple being positive for the allele. Table 4 shows odds ratios (ORs) of HPV infection concordance for the latter 2 categories relative to complete absence of the allele in the couple for HLA-B*07, -DRB1, and -DQB1 alleles. Similarly, Table 5 shows the equivalent results for HLA-G alleles and 3ʹUTR SNPs. A few entries in both tables indicated significant associations between allele sharing and concordance (2 in Table 4 and 5 in Table 5). However, given the numbers of associations examined in Table 4 (22 alleles × 4 HPV concordance outcomes × 2 sharing levels = 176 ORs) and in Table 5 (similar calculation for 15 alleles = 120 ORs), the 7 flagged associations could be due to chance. Moreover, no pattern emerged for the few identified associations. In the interest of conservatism, we repeated the analyses shown in Tables 4 and 5 for selected alleles but by rearranging the partner genetic categories as (1) allele not present in either partner or in 1 partner only; (2) presence in both partners (both heterozygous or 1 heterozygous and the other homozygous); (3) both partners homozygous. Again, no patterns of association emerged that could not be due to chance (Supplementary Table 1).

**Table 4.** Association Between Within-Couple Sharing of Human Leukocyte Antigen B*07, DRB1, and DQB1 Alleles and Human Papillomavirus (HPV) Type Concordance by Subgenera Among the 271 Heterosexual Couples That Were HPV Positive

		OR (95% CI)			
HLA Allele	Level of Allele Sharing^a^	All HPVs	Subgenus 1	Subgenus 2	Subgenus 3
B*07	0	1.0	1.0	1.0	1.0
	1	1.07 (.74–1.55)	1.12 (.53–2.39)	1.13 (.72–1.76)	0.82 (.43–1.56)
	2	2.52 (.74–1.55)	2.74 (.24–31.47)	2.59 (.85–7.88)	3.18 (.34–29.34)
DRB1*01:01	0	1.0	1.0	1.0	1.0
	1	1.00 (.63–1.60)	0.93 (.35–2.50)	0.86 (.49–1.52)	1.42 (.62–3.24)
	2	2.57 (.36–18.32)	1.28 (.08–21.02)	ND	ND
DRB1*03:01	0	1.0	1.0	1.0	1.0
	1	1.46 (.98–2.17)	1.42 (.62–3.27)	1.28 (.80–2.03)	1.65 (.83–3.29)
	2	1.23 (.46–3.29)	1.40 (.09–23.05)	1.11 (.35–3.54)	1.20 (.30–4.79)
DRB1*04:01	0	1.0	1.0	1.0	1.0
	1	1.02 (.66–1.59)	0.45 (.19–1.05)	1.10 (.66–1.86)	1.66 (.74–3.75)
	2	1.17 (.21–6.59)	NC	1.24 (.08–19.98)	1.77 (.16–20.19)
DRB1*04:04	0	1.0	1.0	1.0	1.0
	1	1.12 (.63–2.02)	0.29 (.06–1.42)	1.20 (.60–2.38)	1.80 (.57–5.70)
	2	…	…	…	…
DRB1*07:01	0	1.0	1.0	1.0	1.0
	1	0.82 (.57–1.17)	1.14 (.56–2.33)	**0.63 (.41–.96)**	1.02 (.55–1.90)
	2	0.96 (.45–2.05)	0.91 (.15–5.75)	0.73 (.30–1.80)	1.48 (.40–5.44)
DRB1*11:01	0	1.0	1.0	1.0	1.0
	1	1.13 (.75–1.70)	1.55 (.70–3.43)	1.05 (.65–1.70)	1.09 (.55–2.19)
	2	3.51 (.36–34.27)	ND	ND	ND
DRB1*11:04	0	1.0	1.0	1.0	1.0
	1	1.14 (.73–1.80)	0.96 (.38–2.45)	1.22 (.70–2.11)	1.06 (.52–2.20)
	2	ND	ND	ND	ND
DRB1*15:01	0	1.0	1.0	1.0	1.0
	1	1.30 (.90–1.88)	1.35 (.62–2.94)	1.51 (.97–2.34)	1.12 (.60–2.10)
	2	0.89 (.31–2.52)	0.44 (.09–2.19)	1.46 (.41–5.18)	ND
DRB1*16:01	0	1.0	1.0	1.0	1.0
	1	0.79 (.43–1.47)	1.12 (.36–3.49)	0.65 (.30–1.44)	1.12 (.60–2.10)
	2	2.34 (.37–14.80)	ND	5.25 (.48–57.42)	ND
DQB1*02:01	0	1.0	1.0	1.0	1.0
	1	1.21 (.82–1.80)	1.09 (.48–2.50)	1.15 (.73–1.83)	1.27 (.64–2.51)
	2	1.03 (.36–2.98)	1.32 (.08–21.72)	1.04 (.30–3.67)	0.87 (.20–3.73)
DQB1*02:02	0	1.0	1.0	1.0	1.0
	1	1.01 (.69–1.48)	1.26 (.60–2.66)	0.80 (.51–1.27)	1.17 (.61–2.23)
	2	0.81 (.36–1.80)	0.83 (.19–3.66)	0.73 (.27–1.97)	0.86 (.26–2.87)
DQB1*03:01	0	1.0	1.0	1.0	1.0
	1	0.98 (.67–1.43)	0.98 (.46–2.09)	0.99 (.63–1.55)	1.05 (.56–1.99)
	2	0.63 (.33–1.19)	1.36 (.41–4.44)	0.58 (.27–1.22)	0.50 (.15–1.70)
DQB1*03:02	0	1.0	1.0	1.0	1.0
	1	0.77 (.52–1.14)	0.71 (.33–1.52)	0.79 (.49–1.26)	0.89 (.45–1.75)
	2	0.79 (.30–2.05)	ND	1.19 (.41–3.47)	0.52 (.08–3.26)
DQB1*03:03	0	1.0	1.0	1.0	1.0
	1	0.85 (.52–1.41)	1.60 (.55–4.66)	0.76 (.42–1.36)	0.79 (.33–1.88)
	2	ND	ND	ND	ND
DQB1*04:02	0	1.0	1.0	1.0	1.0
	1	1.01 (.58–1.78)	0.82 (.26–2.63)	1.37 (.71–2.66)	0.57 (.23–1.43)
	2	1.15 (.14–9.27)	ND	1.26 (.08–20.25)	ND
DQB1*05:01	0	1.0	1.0	1.0	1.0
	1	1.12 (.77–1.64)	1.08 (.51–2.29)	0.97 (.61–1.52)	1.64 (.85–3.15)
	2	1.14 (.31–4.17)	0.65 (.06–7.49)	1.93 (.40–9.39)	0.47 (.04–5.45)
DQB1*05:02	0	1.0	1.0	1.0	1.0
	1	1.03 (.57–1.87)	1.59 (.51–4.95)	0.83 (.39–1.76)	0.57 (.23–1.43)
	2	2.40 (.38–15.20)	ND	5.36 (.49–58.90)	ND
DQB1*05:03	0	1.0	1.0	1.0	1.0
	1	1.34 (.70–2.56)	0.91 (.25–3.28)	1.09 (.51–2.32)	2.93 (.93–9.22)
	2	1.41 (.34–5.85)	3.99 (.43–37.01)	0.95 (.17–5.16)	0.90 (.57–14.36)
DQB1*06:02	0	1.0	1.0	1.0	1.0
	1	1.09 (.75–1.60)	1.15 (.52–2.53)	1.19 (.76–1.86)	1.05 (.55–2.00)
	2	1.04 (.48–2.26)	0.53 (.13–2.11)	1.69 (.66–4.31)	0.83 (.20–3.52)
DQB1*06:03	0	1.0	1.0	1.0	1.0
	1	0.82 (.47–1.44)	0.74 (.23–2.40)	0.95 (.48–1.87)	0.72 (.29–1.82)
	2	0.76 (.29–1.99)	0.40 (.08–1.99)	0.80 (.23–2.85)	1.70 (.29–9.98)
DQB1*06:04	0	1.0	1.0	1.0	1.0
	1	1.32 (.78–2.25)	1.09 (.43–2.76)	1.85 (.98–3.49)	0.77 (.26–2.24)
	2	0.77 (.12–4.91)	ND	**0.79 (.11–.96)**	ND

Significant ORs are shown in bold.

Abbreviations: CI, confidence interval; HLA, human leukocyte antigen; HPV, human papillomavirus; ND, not determined; OR, odds ratio.

a 0, allele not present in either partner; 1, presence in 1 partner; 2, presence in both partners.

**Table 5.** Association Between Within-Couple Sharing of Human Leukocyte Antigen G Alleles and 3ʹ Untranslated Region Single-Nucleotide Polymorphisms and Human Papillomavirus (HPV) Type Concordance by Subgenera Among the 271 Heterosexual Couples That Were HPV Positive

		OR (95% CI)			
HLA-G Allele/Variant	Level of Allele Sharing^a^	All HPVs	Subgenus 1	Subgenus 2	Subgenus 3
01:01:01	0	1.0	1.0	1.0	1.0
	1	0.83 (.30–2.28)	0.86 (.38–1.96)	0.85 (.24–2.94)	0.40 (.07–2.32)
	2	0.84 (.31–2.32)	ND	0.71 (.20–2.45)	0.97 (.17–5.68)
01:01:02	0	1.0	1.0	1.0	1.0
	1	1.01 (.69–1.47)	**3.29 (1.52–7.15)**	0.79 (.50–1.24)	0.95 (.50–1.81)
	2	0.84 (.47–1.51)	2.07 (.63–6.80)	0.74 (.37–1.49)	0.70 (.26–1.87)
01:01:03	0	1.0	1.0	1.0	1.0
	1	**0.55 (.35–.87)**	ND	0.65 (.38–1.12)	0.36 (.12–1.03)
	2	1.23 (.80–1.18)	ND	0.67 (.10–4.62)	ND
01:03	0	1.0	1.0	1.0	1.0
	1	1.03 (.64–1.65)	0.71 (.26–1.91)	1.01 (.58–1.78)	1.24 (.54–2.85)
	2	…	…	…	…
01:04:01	0	1.0	1.0	1.0	1.0
	1	0.97 (.66–1.42)	0.57 (.26–1.25)	1.21 (.77–1.90)	0.70 (.37–1.34)
	2	0.83 (.36–1.91)	0.27 (.03–2.41)	1.20 (.45–3.22)	0.59 (.17–2.06)
01:06	0	1.0	1.0	1.0	1.0
	1	0.98 (.60–1.62)	0.49 (.22–1.09)	0.96 (.52–1.77)	0.67 (.30–1.50)
	2	0.89 (.30–2.62)	ND	1.09 (.33–3.63)	1.16 (.19–7.26)
14 bp	0	1.0	1.0	1.0	1.0
	1	1.05 (.62–1.80)	1.20 (.43–3.38)	0.98 (.51–1.87)	0.96 (.37–2.49)
	2	0.73 (.42–1.26)	1.81 (.62–5.26)	0.55 (.28–1.06)	0.83 (.56–3.16)
+3001 C/T	0	1.0	1.0	1.0	1.0
	1	2.21 (.91–5.34)	4.08 (.87–19.15)	1.85 (.71–4.84)	1.69 (.30–9.59)
	2	0.92 (.22–3.82)	ND	0.81 (.19–3.51)	ND
+3003 T/C	0	1.0	1.0	1.0	1.0
	1	1.35 (.93–1.95)	1.05 (.51–2.14)	1.40 (.91–2.17)	1.61 (.81–3.18)
	2	0.50 (.19–1.33)	1.32 (.08–21.95)	0.40 (.13–1.24)	0.94 (.13–6.93)
+3010 G/C	0	1.0	1.0	1.0	1.0
	1	0.99 (.68–1.42)	1.85 (.91–3.76)	1.13 (.73–1.74)	**0.49 (.26–.94)**
	2	1.23 (.62–2.44)	1.88 (.47–7.54)	1.22 (.54–2.76)	0.90 (.27–3.05)
+3027 C/A	0	1.0	1.0	1.0	1.0
	1	0.69 (.39–1.21)	0.74 (.21–2.67)	0.86 (.45–1.65)	0.40 (.14–1.17)
	2	1.32 (.22–7.97)	ND	0.71 (.11–4.80)	ND
+3035 C/T	0	1.0	1.0	1.0	1.0
	1	0.77 (.48–1.24)	0.97 (.32–2.96)	0.76 (.44–1.32)	0.80 (.34–1.92)
	2	0.99 (.28–3.55)	2.78 (.25–30.88)	0.80 (.22–2.96)	ND
+3142 C/G	0	1.0	1.0	1.0	1.0
	1	0.98 (.61–1.57)	1.53 (.62–3.78)	0.98 (.55–1.74)	0.66 (.29–1.54)
	2	0.89 (.56–1.40)	1.49 (.60–3.66)	1.03 (.60–1.78)	0.48 (.21–1.08)
+3187 G/A	0	1.0	1.0	1.0	1.0
	1	1.02 (.49–2.11)	0.61 (.17–2.23)	1.89 (.76–4.69)	0.14 (.02–1.18)
	2	0.95 (.47–1.90)	1.14 (.34–3.87)	1.51 (.64–3.58)	0.14 (.02–1.18)
+3196 C/G	0	1.0	1.0	1.0	1.0
	1	0.96 (.65–1.43)	**2.58 (1.18–5.66)**	0.96 (.60–1.53)	0.63 (.32–1.24)
	2	1.10 (.66–1.81)	**4.23 (1.52–11.76)**	0.93 (.51–1.69)	0.78 (.34–1.79)

Significant ORs are shown in bold.

Abbreviations: CI, confidence interval; HLA, human leukocyte antigen; HPV, human papillomavirus; ND, not determined; OR, odds ratio.

a 0, allele not present in either partner; 1, presence in 1 partner; 2, presence in both partners.

Finally, we evaluated the extent of HLA sharing irrespective of HLA class, grouped or in combination, and HPV concordance between the dyads (Table 6). There was no discernible pattern or trend of association between the extent of allele sharing and HPV type concordance, and the 5 entries in the table that were significant at the 5% level could have been due to chance, owing to the high number of associations examined.

**Table 6.** Associations Between Extent of Human Leukocyte Antigen Allele Sharing and Human Papillomavirus (HPV) Concordance Among the 271 Heterosexual Couples That Were HPV Positive

		Age-Adjusted OR (95% CI)^a^			
Inclusiveness of HLA Groupings Shared	Alleles Shared per Couple	All HPVs	Subgenus 1	Subgenus 2	Subgenus 3
HLA-B*07, -DRB1, and -DQB1	0	1.0	1.0	1.0	1.0
	1	1.1 (.8–1.7)	0.9 (.4–2.1)	1.3 (.8–2.2)	0.9 (.4–1.9)
	2–3	1.0 (.6–1.6)	0.8 (.3–2.2)	1.3 (.7–2.5)	0.6 (.2–1.5)
HLA-G	0	1.0	1.0	1.0	1.0
	1	1.1 (.7–1.6)	**0.4 (.2–.7)**	1.1 (.7–1.7)	**1.9 (1.0–3.5)**
	2	0.6 (.3–1.5)	0.6 (.1–2.7)	0.4 (.1–1.1)	3.0 (.5–16.7)
All HLAs	0	1.0	1.0	1.0	1.0
	1	1.6 (.7–1.9)	0.7 (.2–1.8)	1.1 (.6–2.0)	1.5 (.7–3.3)
	2	1.0 (.6–1.7)	0.8 (.3–2.4)	0.9 (.5–1.8)	1.2 (.5–2.9)
	3–5	1.1 (.5–2.1)	**0.2 (.0–1.0)**	1.6 (.7–3.7)	1.4 (.4–4.7)
HLA-G 3ʹUTR: 14 bp, +3142 C/G, +3187 G/A	0	1.0	1.0	1.0	1.0
	1	0.9 (.6–1.5)	1.4 (.6–3.5)	0.6 (.4–1.2)	**2.4 (1.0–5.7)**
	2	0.8 (.5–1.3)	0.9 (.3–2.5)	0.9 (.5–1.6)	0.8 (.4–1.9)
	3	0.7 (.4–1.2)	2.4 (.9–6.3)	0.6 (.3–1.1)	0.8 (.4–1.8)
HLA-G 3ʹUTR: 14 bp and all 8 different SNPs	0	1.0	1.0	1.0	1.0
	1	1.0 (.6–1.6)	1.5 (.6–3.7)	0.7 (.4–1.3)	**2.6 (1.0–6.3)**
	2	0.6 (.4–1.1)	0.8 (.3–2.5)	0.7 (.4–1.3)	0.7 (.3–1.7)
	3	0.7 (.3–1.2)	1.0 (.2–4.9)	0.6 (.3–1.2)	0.7 (.2–2.0)
	4	0.9 (.5–1.6)	2.4 (.8–7.3)	0.8 (.4–1.5)	0.8 (.3–1.9)
	5–6	0.9 (.3–2.5)	6.9 (.9–54.6)	0.4 (.1–1.4)	4.0 (.4–36.9)

Significant ORs are shown in bold.

a Results according to inclusiveness of HLA classification.

Abbreviations: CI, confidence interval; HLA, human leukocyte antigen; HPV, human papillomavirus; OR, odds ratio; SNP, single-nucleotide polymorphism; UTR, untranslated region.

## DISCUSSION

The role of different host factors in transmission of HPV infections between heterosexual couples is not well known. Since antigen processing as the initiating step in immune response requires mediation via HLA [8], we hypothesized that HLA allele sharing between partners would facilitate transmission of HPV infections within heterosexual couples. Our HITCH cohort of young couples provides a suitable observational study design to study the role of HLA polymorphism on HPV transmission. As a strength, all HITCH couples had recently initiated their sexual relationship, when most HPV transmission episodes are known to occur [18, 19]. We also had high-resolution typing for 106 different HLA alleles investigated with substantial (48%) between-partner type-specific HPV concordance, our surrogate endpoint for considering that transmission had occurred.

We did not find evidence to support our hypothesis. Allele sharing, individually or in combination over multiple loci, was not statistically associated (beyond chance expectation) with presumed infection transmission using different HPV type groupings chosen according to biological behavior and pathogenetic propensity.

HLA genetic variation has been demonstrated to affect risk of HPV-related cervical cancer, especially with HLA class I and II loci molecules [6, 20–22]. Deletions in HLA-B alleles are associated with cervical carcinogenesis [6, 22]. A recent meta-analysis found the class II alleles HLA-DQR*02, -*03, and -*06:03 to decrease and HLA-DQB1*05, -*03:01, and -*04:02 to increase risk of cervical cancer [20], while a genome-wide study concluded HLA-DRB1*06:02 and -*15:01 to be major risk alleles in cervical carcinogenesis [6]. None of the above class I and II HLA alleles were associated with HPV transmission in our couples study.

The nonclassical class Ib HLA-G molecules have been suggested to play a prognostic or risk marker role because of HLA-G alleles’ relatively low polymorphism rate and restricted tissue distribution compared to other HLA molecules [23]. Different HLA-G alleles were shown to have a distinctive role in the reproductive system of females and males [24, 25] and also in other cancers and infections by facilitating their escape from immune surveillance [26]. HLA-G was shown to be important in mother-to-child transmission of HIV infection, mainly because of its preferential expression at the maternal-fetal interface and its immunosuppressive properties [10, 27–29]. A recent study investigated the role of HLA-G alleles in the vertical transmission of HPV infection but could not confirm HLA-G allele sharing to impact the HPV transmission between mother and child at birth or perinatally [11]. Few studies showed HLA-G*01:01:01, -*01:01:03, and -*01:01:05 and the HLA-G 14bp deletion to be associated with HPV infection or cervical cancer among women [15, 30–32]. Our results could not confirm HLA-G alleles to mediate risk of HPV transmission between partners.

In conclusion, our results do not support a role for HLA allele sharing in influencing transmission of genital HPV infection. HPV transmission within heterosexual couples is likely to be a more complex combination of host and environmental factors.

## Supplementary Data

Supplementary materials are available at *The Journal of Infectious Diseases* online. Supplementary materials consist of data provided by the author that are published to benefit the reader. The posted materials are not copyedited. The contents of all supplementary data are the sole responsibility of the authors. Questions or messages regarding errors should be addressed to the author.

jiac115_suppl_Supplementary_Table_S1

## Notes

***Acknowledgments.*** The authors are indebted to all of the participants in the HITCH Cohort Study. The authors also acknowledge Allita Rodrigues (study coordination); Gail Kelsall, Suzanne Dumais, Natalia Morykon, and Amelia Rocamora (management of subject participation and specimen collection); Nathalie Slavtcheva (study management); Veronika Moravan (data management); Hélène Voyer, Véronique Legault, Emilie Comete, and Julie Guénoun (human papillomavirus [HPV] testing); and Karine Beauchemin, Marie-Claude Faucher, and Annie Jeanneau (human leukocyte antigen testing).

***Financial support.*** This study was funded by the Canadian Institutes of Health Research (grant numbers MOP-68893 and CRN-83320); the US National Institutes of Health (grant number AI073889); Merck & Co; and Réseau Fonds de recherche du Québec, Réseau sida et maladies infectieuses. K. L. has received research funding from the Sigrid Juselius Foundation and the Finnish Medical Foundation.
