# Supplementary material for: Role of Human Leukocyte Antigen Allele Sharing in Human Papillomavirus Infection Transmission Among Heterosexual Couples: Findings From the HITCH Cohort Study
Source: J Infect Dis. 2022 Apr 1;226(7):1175–83. doi: 10.1093/infdis/jiac115 (PMC9518836; doi:10.1093/infdis/jiac115)
Supplement: jiac115_suppl_Supplementary_Table_S1 [file jiac115_suppl_supplementary_table_s1.docx]

**Supplementary Table 1.** Association between within-couple sharing of HLA-B*07, DRB1, DQB1 and G alleles and HPV-type concordance by group among the 271 heterosexual couples that were HPV positive.

| HLA allele | Level* of Allele sharing | Odds ratio (95% confidence interval) | | | |
| --- | --- | --- | --- | --- | --- |
|  |  | All HPVs | Subgenus 1 | Subgenus 2 | Subgenus 3 |
| B*07 | 0 | 1.0 | 1.0 | 1.0 | 1.0 |
|  | 1 | 2.46 (0.93-6.48) | 2.64 (0.23-29.86) | 2.49 (0.82-7.48) | 3.39 (0.37-31.01) |
|  | 2 | … | … | … | … |
| DRB1*01:01 | 0 | 1.0 | 1.0 | 1.0 | 1.0 |
|  | 1 | 2.57 (0.36-18.29) | 1.29 (0.08-21.15) | ND | ND |
|  | 2 | **…** | **…** | **…** | **…** |
| DRB1*03:01 | 0 | 1.0 | 1.0 | 1.0 | 1.0 |
|  | 1 | 1.10 (0.42-2.93) | 1.29 (0.08-21.15) | 1.03 (0.32-3.27) | 1.04 (0.26-4.05) |
|  | 2 | **…** | **…** | **…** | **…** |
| DRB1*04:01 | 0 | 1.0 | 1.0 | 1.0 | 1.0 |
|  | 1 | 1.16 (0.21-6.54) | ND | 1.21 (0.08-19.54) | 1.64 (0.15-18.53) |
|  | 2 | **…** | **…** | **…** | **…** |
| DRB1*04:04 | 0 | 1.0 | 1.0 | 1.0 | 1.0 |
|  | 1 | **…** | **…** | **…** | **…** |
|  | 2 | **…** | **…** | **…** | **…** |
| DRB1*07:01 | 0 | 1.0 | 1.0 | 1.0 | 1.0 |
|  | 1 | 1.06 (0.50-2.22) | 0.86 (0.14-5.25) | 0.90 (0.36-2.24) | 1.47 (0.41-5.21) |
|  | 2 | **…** | **…** | **…** | **…** |
| DRB1*11:01 | 0 | 1.0 | 1.0 | 1.0 | 1.0 |
|  | 1 | 3.40 (0.35-33.19) | ND | ND | ND |
|  | 2 | **…** | **…** | **…** | **…** |
| DRB1*11:04 | 0 | 1.0 | 1.0 | 1.0 | 1.0 |
|  | 1 | () | () | () | () |
|  | 2 | **…** | **…** | **…** | **…** |
| DRB1*15:01 | 0 | 1.0 | 1.0 | 1.0 | 1.0 |
|  | 1 | 0.81 (0.28-2.30) | 0.40 (0.08-1.97) | 1.25 (0.35-4.41) | () |
|  | 2 | **…** | **…** | **…** | **…** |
| DRB1*16:01 | 0 | 1.0 | 1.0 | 1.0 | 1.0 |
|  | 1 | 3.76 (0.31-46.20) | ND | 3.97 (0.33-47.48) | ND |
|  | 2 | 1.16 (0.06-21.99) | ND | ND | ND |
| DQB1*02:01 | 0 | 1.0 | 1.0 | 1.0 | 1.0 |
|  | 1 | 0.97 (0.34-2.80) | 1.29 (0.08-21.15) | 1.00 (0.29-3.49) | 0.82 (0.19-3.42) |
|  | 2 | **…** | **…** | **…** | **…** |
| DQB1*02:02 | 0 | 1.0 | 1.0 | 1.0 | 1.0 |
|  | 1 | 0.81 (0.37-1.77) | 0.75 (0.18-3.22) | 0.79 (0.29-2.09) | 0.81 (0.25-2.65) |
|  | 2 | **…** | **…** | **…** | **…** |
| DQB1*03:01 | 0 | 1.0 | 1.0 | 1.0 | 1.0 |
|  | 1 | 0.65 (0.35-1.21) | 1.60 (0.52-4.95) | 0.58 (0.28-1.20) | 0.48 (0.15-1.56) |
|  | 2 | 0.37 (0.03-5.27) | ND | 0.57 (0.04-8.41) | ND |
| DQB1*03:02 | 0 | 1.0 | 1.0 | 1.0 | 1.0 |
|  | 1 | **…** | **…** | **…** | **…** |
|  | 2 | **…** | **…** | **…** | **…** |
| DQB1*03:03 | 0 | 1.0 | 1.0 | 1.0 | 1.0 |
|  | 1 | 0.85 (0.52-1.41) | 1.60 (0.55-4.66) | 0.76 (0.43-1.36) | 0.78 (0.33-1.88) |
|  | 2 | ND | ND | ND | ND |
| DQB1*04:02 | 0 | 1.0 | 1.0 | 1.0 | 1.0 |
|  | 1 | 1.15 (0.14-9.25) | ND | 1.21 (0.08-19.54) | ND |
|  | 2 | **…** | **…** | **…** | **…** |
| DQB1*05:01 | 0 | 1.0 | 1.0 | 1.0 | 1.0 |
|  | 1 | 1.10 (0.30-4.00) | 0.64 (0.06-7.21) | 1.95 (0.40-9.42) | 0.40 (0.04-4.59) |
|  | 2 | **…** | **…** | **…** | **…** |
| DQB1*05:02 | 0 | 1.0 | 1.0 | 1.0 | 1.0 |
|  | 1 | 3.76 (0.31-46.20) | ND | 3.97 (0.33-47.48) | ND |
|  | 2 | 1.16 (0.06-21.99) | ND | ND | ND |
| DQB1*05:03 | 0 | 1.0 | 1.0 | 1.0 | 1.0 |
|  | 1 | 1.38 (0.33-5.77) | 4.02 (0.43-37.18) | 0.94 (0.17-5.13) | 0.82 (0.05-13.64) |
|  | 2 | **…** | **…** | **…** | **…** |
| DQB1*06:02 | 0 | 1.0 | 1.0 | 1.0 | 1.0 |
|  | 1 | 1.00 (0.47-2.17) | 0.51 (0.13-2.00) | 1.58 (0.63-3.96) | 0.82 (0.20-3.40) |
|  | 2 | **…** | **…** | **…** | **…** |
| DQB1*06:03 | 0 | 1.0 | 1.0 | 1.0 | 1.0 |
|  | 1 | 0.78 (0.30-2.03) | 0.41 (0.08-2.05) | 0.81 (0.23-2.86) | 1.75 (0.30-10.15) |
|  | 2 | **…** | **…** | **…** | **…** |
| DQB1*06:04 | 0 | 1.0 | 1.0 | 1.0 | 1.0 |
|  | 1 | 0.75 (0.12-4.76) | ND | 0.73 (0.10-5.52) | ND |
|  | 2 | **…** | **…** | **…** | **…** |
| G*01:01:01 | 0 | 1.0 | 1.0 | 1.0 | 1.0 |
|  | 1 | 0.93 (0.65-1.33) | **0.47 (0.24-0.95)** | 0.73 (0.48-1.11) | 2.24 (1.18-4.23) |
|  | 2 | 2.31 (0.92-5.82) | 0.66 (0.14-3.17) | 4.51 (1.14-17.89) | 2.91 (0.53-15.90) |
| G*01:01:02 | 0 | 1.0 | 1.0 | 1.0 | 1.0 |
|  | 1 | 0.84 (0.49-1.44) | 0.98 (0.32-2.98) | 0.85 (0.44-1.63) | 1.72 (0.29-1.80) |
|  | 2 | … | … | ... | … |
| G*01:01:03 | 0 | 1.0 | 1.0 | 1.0 | 1.0 |
|  | 1 | 1.38 (0.23-8.17) | ND | 0.72-0.11-4.83) | ND |
|  | 2 | … | …. | … | … |
| G*01:03 | 0 | 1.0 | 1.0 | 1.0 | 1.0 |
|  | 1 | … | … | … | … |
|  | 2 | … | … | … | … |
| G*01:04:01 | 0 | 1.0 | 1.0 | 1.0 | 1.0 |
|  | 1 | 0.84 (0.37-1.91) | 0.32 (0.04-2.83) | 1.12 (0.42-2.98) | 0.67 (0.19-2.30) |
|  | 2 | … | … | … | … |
| G*01:06 | 0 | 1.0 | 1.0 | 1.0 | 1.0 |
|  | 1 | 0.89 (0.30-2.62) | ND | 1.09 (0.33-3.65) | 1.25 (0.20-7.76) |
|  | 2 | … | … | … | … |

*0: Allele not present in either partner or in one partner only, 1: Presence in both partners (both heterozygous or one heterozygous and the other homozygous), 2: Both partners homozygous.

ND: not determined
